# Ladies’ Hahnemann Monument Association

**Published:** 1899-03

**Authors:** 


### LADIES' HAHNEMANN MONUMENT ASSOCIATION.

BUFFALO, FEBRUARY 1st, 1899.

EDITOR OF THE HOMŒOPATHIC PHYSICIAN:—The President of the L. H. M. A. has authorized me to communicate some facts which may be of interest to your readers. The work of raising money for the Hahnemann monument, by the above organization, came to an abrupt standstill soon after the association was formed, on account of the war with Spain.

Now that these momentous conditions are changed to an era of peace and prosperity, the project is again being pushed in every part of the United States, of collecting the requisite amount to assist in completing this most superb memorial to the founder of Homœopathy.

Many distinguished women in all sections of the country are interested in this movement. A few names will suffice to show the kind of representation the organization has: Mrs. M. A. Hanna, Ohio; Mrs. George Westinghouse, Washington, D. C.; Mrs. John Dalzell, Pennsylvania; Mrs. James A. Mount, Indiana; Mrs. H. Clay Evans, Tennessee; Mrs. William Appleton, Massachusetts; Mrs. H. N. Higinbotham, Illinois; Mrs. John S. Newberry, Michigan; Mrs. Elihu Root, New York; Mrs. John H. Vincent, Kansas.

Could this work of raising a monument to Samuel Hahnemann have a stronger endorsement among the laity than these brilliant names?

This effort, combined with the splendid achievement of the physicians' committee in obtaining $30,000 for this object insures the success of the entire movement.

One of the several methods employed by the L. H. M. A. has been to commence sending out a personal letter to each homœopathic physician, intending to interest those who have not yet given, asking for small contributions to the fund.

This appeal, only just begun, has, at the outset, met with most encouraging results, especially noteworthy because many of the physicians whose names are here given had already contributed once, twice, and even thrice, to the Hahnemann Monument Fund. The courtesy of the replies, for promptness, kind words, and the enclosures is deeply appreciated by all concerned.

The following physicians have contributed to February 1st, not including contributions from the "Chain Method," commenced in the autumn:

| | |
|---|---|
| Herbert A. Sherwood, M. D., | $5 00 |
| Arthur F. Bissell, M. D.,... | 5 00 |
| J. G. Baldwin, M. D.,...... | 2 00 |
| Daniel H. Arthur, M. D.,... | 2 00 |
| John Arschagouni, M. D.,.. | 2 00 |
| P. L. Hatch, M. D.,....... | 3 00 |
| F. P. Batcheldor, M. D., and wife, .................... | 2 00 |
| George A. Adams, M. D., .. | 2 00 |
| C. F. Barber, M. D., ....... | 2 00 |
| F. J. Becker, M. D.,........ | 3 00 |
| Chas. P. Beaman, M. D.,... | 2 00 |
| A. B. Berghaus, M. D., .... | 2 00 |
| Joseph P. Paine, M. D.,.... | 2 00 |
| Francis M. Bennett, M. D.,. | $2 00 |
| A. B. Blackman, M. D.,.... | 1 00 |
| J. Arthur Bullard, M. D.,... | 2 00 |
| Merritt C. Bragdon, M. D., . | 2 00 |
| J. D. Brewster, M. D.,..... | 4 00 |
| J. P. Bloss, M. D.,......... | 5 00 |
| A. J. Bond, M. D.,......... | 2 00 |
| F. C. Bowman, M. D.,...... | 2 00 |
| Herbert M. Bishop, M. D.,. | 1 00 |
| H. F. Biggar, M. D.,....... | 25 00 |
| J. D. Burns, M. D.,........ | 2 00 |
| | $82 00 |

In the near future the complete report of the Treasurer, Mrs. A. R. Wright, will be forwarded for publication in your valuable pages. Physicians' contributions will be sent as often as amounts warrant it.

It is very encouraging to know that medical societies are taking up the Hahnemann Monument matter, as the following letter will show:

BOSTON, JANUARY 10th, 1899.

"DEAR MRS. COOK:—The work of the Ladies' Hahnemann Monument Association, as outlined in yours of December 31st, and in the enclosed circular, was presented at the annual meeting of our Boston Homœopathic Society, held last Thursday evening, January 5th.

"The whole subject of the monument, past and present efforts, was discussed with much interest by various members. It was voted to appoint a committee who should have the power to take the necessary steps to secure the co-operation of the members to raise among the laity, as well as the profession of Boston, a sum that will aggregate at least $1,000.

"Drs. A. J. Baker Flint, Adeline B. Church, Lucy Appleton, and Sarah S. Windsor, President-elect, were appointed such committee.

"The movement instituted by the L. H. M. A. must prove a grand success. I also take pleasure in informing you that another start has been made by the present students of Boston University School of Medicine, who do not wish to be left behind in this movement to the honor of Hahnemann and Homœopathy.

"It is of added significance that the leader in this and the Editor-in-chief of the College paper, *The Medical Student*, is a lady, Miss Alberta S. Boomhower.

"The movement is an infectious one, and may it spread and spread, until success crowns the faithful efforts put forth.

"Very sincerely yours,

"BOSTONIAN M. D."

It is earnestly hoped that medical societies everywhere will take up this matter and co-operate with the L. H. M. A. to interest the laity in paying this tribute to the greatest reformer of the century.

The following physicians' wives are identified with the central organization at Buffalo, N. Y.:

Mrs. Joseph T. Cook, President; Mrs. F. Park Lewis, Vice-President; Mrs. Burt J. Maycock, Vice-President; Mrs. Dewitt G. Wilcox, Vice-President; Mrs. E. P. Hussey, Vice-President; Mrs. Wm. Henry Marcy, Vice-President; Mrs. Hubbard A. Foster, Mrs. P. A. McCrea, Mrs. John Miller, Advisory Committee; Mrs. A. R. Wright, Treasurer.

Very cordially yours,

ANNIE H. FROST, *Assistant Secretary.*

